# Treatment of Diphtheria

**Published:** 1877-10

**Authors:** E. N. Chapman

**Affiliations:** Late Professor of Obstetrics, etc., L. I. College Hospital


BUFFALO

### Medical and Surgical Journal.

VOL. XVII. OCTOBER, 1877. No. 3.

### Original Communications.

:o:

ART. I.—*Treatment of Diphtheria.* By E. N. CHAPMAN, A. M., M. D., late Professor of Obstetrics, etc., L. I. College Hospital.

The treatment of diphtheria has, the last ten years, been so thoroughly and exhaustively discussed, that it would seem the height of folly, at this late day, to question the matured opinions of practitioners and writers the world over. Nevertheless, from the fatality of this deadly scourge, as it is universally considered, I am induced to offer my experience in a plan of medication which has, after a trial of more than fifteen years, been crowned with a success that throws every other, however pretentious, into the shade. To substantiate this bold statement I shall appeal for proof to the records of the Board of Health of this city, a set of books as certain to dissipate groundless assumptions as to establish sterling facts. The revelations of microscopy, the researches of chemistry, the flights of theory, and the deductions of reason are of little avail in the face of the enemy, when the urgent practical question,—the empirical fact—what will cure the patient, waits a solution.

In 1859 and 1860, whilst employing the means then in vogue, I lost every third or fourth case, a frightful mortality, yet one many physicians bemoan even now; but immediately after instituting an opposite treatment, though the epidemic was at its worst, not more than one in twenty. This treatment, which I have since seen no reason to change, or even to modify in any essential particular, was, together with the cases presented in its support, published in the Boston Medical and Surgical Journal, February 5th, 1863. In this article, reasoning from results to causes, I came to the conclusion that diphtheria is a disease of the blood, a disease tending to rapidly destroy the vitality of that fluid; that the exudation is secondary to this contamination, a local manifestation of a general dyscrasy; that the fever is sympathetic, the turmoil excited by the intrusion of a deadly agent; that alcohol counteracts, neutralizes, or destroys the poison, whatever it may be, acting, in fact, like a true antidote, if promptly and liberally given; that the membrane fails and does not reappear directly the blood fails to offer the proper pabulum for its continual growth; and that bark and iron are the only aids required, aside from substantial food, to conduct ninety-five *per centum* of all cases to a successful issue.

Acting on these principles from December, 1860, to the conclusion of my report, January, 1863, it appears that, of the twenty cases treated with brandy, quinine, and iron, I lost one only, a result so far surpassing what had been attained by others, that I hoped the profession would be induced to give these remedies a fair trial; but, as yet, this hope has not been realized. For this failure, it is easy to allege a sufficient reason. The misfortune of a name often clouds a writer's ideas or, when these are fully grasped, ensures their prompt rejection.

Under ordinary conditions, alcohol is a stimulant; but in diphtheria it lacks this property, a quantity which would in health induce intoxication having no excitant effect: it is thrown off in the breath; but in diphtheria, unless the dose is disproportionately large, no odor is perceptible until the disease begins to yield: it is contraindicated in acute attacks of fever, especially if attended with local inflammation; but in diphtheria the contrary holds true: it is demanded, when the vascular excitement lessens and nervous energy flags, to support the vital forces in the effort to rally from the shock, and regain their supremacy; but in diphtheria the poison has, at this stage of the disease, done its work so thoroughly that alcohol no longer, or but feebly, manifests its antidotal property.

Hence, from the more common action of alcohol, and the more common use to which it is applied, it is well-nigh impossible to gain a hearing, when, on the strength of clinical data alone, one asserts that alcohol in diphtheria is not a stimulant, but a febrifuge; is not an excitant, but a sedative; is not a tonic to support the strength, but an antidote to neutralize the poison; its full efficacy being shown at the outset of the disease when fever is high and inflammation acute. In fact, it is as much a specific for the diphtheritic poison as quinia for the malarial; each breaking up a special morbid process, and rendering a prompt return to health possible, through an unknown potency that has as yet defied the search of science. So, also, the analogy holds as to the prophylactic qualities of these two remedies; what is curative being equally preventative in each instance.

From the treatment I commenced at the close of the year 1859, and published two years later in the Boston Medical and Surgical Journal—a treatment which has to this day made no advance in professional favor—I will make the following extract, inasmuch as it inculcates the therapeutical principles that have since, by a singularly small ratio of mortality, been notably vindicated, if, peradventure, success for a series of years is the touchstone by which to test medical doctrines.

"With a singular uniformity, the stimulating treatment, whether in the acute or chronic stage—that of excitement, fever, and inflammation, or of prostration, rheumatism, or dropsy—had the same happy effect; and it was in all conditions, that had a diphtheritic origin, uninterruptedly followed; since we only regarded the causation, not its manifestations—the root of the evil, not its offshoots—and directed our efforts to the removal of a special state of the blood. This state of the blood, which is prone to occur in scrofulous children, or adults reduced by disease, or of feeble constitutions, in a certain endemic condition of the atmosphere, is marked by a diminished vital power which, being exalted by stimulants, the symptoms are checked, the inflammation subdued, the membrane removed, a rapid recovery effected, and relapses prevented.

In other words, this plan of medication is radical, strikes at the heart of the trouble; whereas most others that have been proposed are but an ineffectual warfare against symptoms. The blood, which is similarly affected in the mild or severe cases, in the first or later stages, only differing in the degree of its dissolution, alone claims our attention. Against this condition, before the disintegration is irreparable, we bring to bear the most powerful means in our hands, to buoy up the constitutional powers, and sustain the activity and energy of each function. The first link in this morbid chain being this retrograde movement in the vitality of the blood, when this is checked, fever, inflammation, hæmorrhage, exudation, collapse, paralysis, dropsy, &c., disappear almost magically, from the simple fact that the cause has been rendered null and inoperative, and the prime pathological change removed.

"Of the remedies that have been employed in diphtheria, two only have proved themselves in my hands worthy of confidence, with the exception, in the chronic stage, in favor of the salts of iron. These two remedies, alcohol or cinchona in one of its forms, are administered in such doses and at such intervals as to secure one effect—the fullest stimulation of the nervous and vascular systems. Either, singly, may suffice when the vital force needs but slight aid to maintain the integrity of the blood; but the two united have more than a double power, and call out the greatest possible amount of resistance, since the nerve-centres and blood-vessels—the great life-factors—are exalted to the highest point. Alcoholic liquors, when given in such quantities and at such intervals as to occasion and keep up a steady but not excessive excitation, not only quicken the functional offices of each organ, but act more especially on the nervous and vascular systems. They bring out the latent powers, arousing them when dormant, and freeing them when oppressed by a load of morbific influences; and thus give, for the time being, the greatest energy to the entire organism. Herein, according to the views of many therapeutists, alone consists the value of this class of stimulants in any disease. The patient lives over the crisis, or the poison is spent or eliminated; and thus recovery becomes possible. This is but a partial estimate of the remedial action of alcohol, which not only produces the effects just mentioned, but others of much greater importance in the present disease—the increased vitality of the blood itself. It is well known that the habitual use of spirituous beverages augments the blood-making process, renders the blood richer in all of its important constituents—the red globules, albumen and fibrin—and of a greater crasis; by which means, there arises an excess of organizable material, that often occasions inflammatory diseases in *bonvivants*. This condition is the opposite to that existing in the diphtheritic subject, whose blood has, invariably, been rendered poor by exhausting disease, or impoverished by the demands of increase and growth, as in the instance of children. These causes are intensified and rendered operative by a scrofulous or syphilitic taint.

"It is a noteworthy fact, that, in my experience, diphtheria never attacks those habituated to the use of spirits. This, if confirmed, may be more than a remarkable coincidence.

I, therefore, from clinical observations and therapeutical deductions, arrive at the practical conclusion, that alcohol is not only a stimulant to the system at large, but also to the blood itself, quickening its vital elaborations, and increasing its vital status, through which a direct barrier is thrown in the way of the disease. In other words, the results produced by the disease, and by the alcohol in the blood, being directly opposite, they neutralize each other; and thus, the stimulant assumes in my eyes the position of a true remedy, a trustworthy antidote. Hence its medicinal power being not only remedial but prophylactic, it will prevent the extension of diphtheria in the other members of the family, as well as cure the one affected. This conclusion is a necessary sequence, if the pathology of diphtheria and the *modus operandi* of alcohol have been correctly appreciated.

In malignant cases of diphtheria, we might desire to avail ourselves of a co-operating remedy—of one, like quinia, that particularly excites the great ganglionic nerve-centres; by which means we should attain a maximum of power, and carry stimulation to the highest possible degree. The various preparations of the cinchona bark fulfil this indication; and, when pushed to the extent of causing *tinnitus aurium*, are our most potent nerve-stimulants. Their efficacy is shown in all diseases when the innervation is weakened, disordered, or perverted; in fevers from malaria, in fevers from a blood-poison, and in a variety of morbid conditions attended with an exhausted or defective nervous energy. As a tenderness of the gums is a mark of the saturation of the system with a mercurial, so the ringing in the ears indicates that the brain is fully under the influence of cinchona. Both it and the alcoholic stimulant, whether used singly or united, should be given with regularity, and in sufficient doses to obtain their full effects; and then the latter, in a lessened quantity, continued for two or more weeks after the disappearance of the disease and its sequelæ. From the outset to a permanent restoration to health, one, or perhaps both of these remedies, are to be continuously administered.

In the more tedious cases that retain a hæmorrhagic tendency, the substitution of a sesqui-salt of iron for the cinchona might, for a time, be advisable, when the peculiar effect of the latter on the brain had been attained. These salts of iron, like the alcohol, increase the crasis and coagulability of the blood, as I have experienced in several instances of internal hæmorrhage; but they affect the body of the blood too slowly to be a trustworthy reliance in acute cases. Their action would be slight, short of two or three days; whereas the progress of diphtheria brooks no delay. Indeed, one of my cases was attacked with the disease, although the persulphate of iron, in free doses, had been in use for hæmoptysis for more than, forty-eight hours. At least fifteen drops of the muriated tincture, or five drops of the solution of the chloride or persulphate of iron, should be administered every third or fourth hour whenever we desire this peculiar change in the blood; but in chronic cases, with more time at our disposal, the dose may be less, since usually our main object is now to remedy the anæmia.

"Most writers insist strongly on the importance of giving large quantities of animal broths to sustain the strength of the patient, and thus enable him to ride out the violence of the disorder. This, as a medicinal means, cannot but be erroneous in the early stages, since most of the patients are taken while eating heartily of animal food, and enjoying their usual health. We could not expect that nourishment, however concentrated, which did not prevent the accession of a disease whilst the digestion was vigorous, would cure it when digestion, assimilation, and nutrition are completely destroyed. The change of food into the living structures is something more than its ingestion into the stomach, or its absorption into the blood-vessels; and nutriment, unappropriated, can be only an incumbrance,—a foreign element—which will be carried off by the kidneys with the effete matters. Most of my patients took little or no nourishment before convalescing, when it was ordered for the same reasons that we order it in other ailments.

"It is important to avoid close, hot, and badly ventilated rooms, and secure a free circulation of air. As soon as practicable, the patient should be taken out of doors, and no fear need be entertained of *catching cold;* the disease having no analogy with tonsillitis, pharyngitis, or any other mucous inflammation whatsoever."

Since writing the above, I have come to rely more and more confidently on alcohol in some one of its many forms, as the remedy *par excellence* not only for diphtheria, but also for all diseases with which it is complicated; for example, croup, scarlatina, pneumonia and albuminuria, and I might say, perhaps, gastritis and meningitis. In fact, diphtheria so contaminates the circulation that recovery is scarcely possible in slighter ailments than these unless this, the major disease, which overrides all else, is promptly attacked by the only agent that will neutralize its venom, and effectually check blood, degeneration. This being done, the case is usually so simplified as speedily to terminate in recovery with little farther aid from art. Often, indeed, these several complications are simply varied expressions of the diphtheritic poison, signs of a general infection of the system, that demand even more urgently the use of stimulants and tonics in full doses.

Still more, diphtheria makes a decided impress, when generally prevalent in a community, on all other diseases by imparting a low type that forbids depressing medication, and demands early stimulation. Antimony, ipecacuanha, and hot baths should in croup soon give place to brandy, quinine, and good food; immediately if the fauces appear at all suspicious, and directly if the reducing plan fails to subdue the rough, rasping cough. Thus the formation of the croupous membrane, which, doubtless, in a certain epidemic state of the air is always of a diphtheritic nature, may be forestalled. So, also, in cases of mucous, follicular, and glandular inflammation of the tonsils, the alcoholic treatment heads off the enemy, should it lie in ambush, and more certainly than any other ensures a prompt and satisfactory recovery. The exudation fully declaring itself, whatever may precede or attend it, the alcohol is to be implicitly relied upon to the exclusion of all else, unless the sulphate of quinia be an exception, and given with the same precision as is requisite to the proper administration of any other important medicine. The conditions of success must be observed scrupulously, as otherwise the alcohol may be robbed of its specific virtues, and the contamination of the blood go on unchecked. Whatever makes a call on the nerves and lessens their tone like cathartics, emetics, and foul air; and whatever deteriorates the blood and impairs its crasis like mercurials, alkalies, and poor food, is to be carefully avoided. Consequently the patient should be in bed, the room cool and well ventilated, evacuants interdicted, the secretions undisturbed, and the fever, local symptoms, or other accessory conditions disregarded. The one indication alone obtains; to wit, the introduction, as promptly as practicable, of a sufficient quantity of alcohol into the circulation to counteract the effects of the poison and prevent morbid changes in the blood. The more speedy the resort to the antidote, the more speedy the cure, a day or two being often sufficient to restore the patient to his usual health; but, if the alcohol be held in reserve until the fever is allayed and the system prepared for stimulants, it will be shorn of its potency, as now the damage has been done and there is no vitality left to respond, though the stomach were flooded with alcohol and stuffed with food. The administration of alcohol, therefore, at the inception of the disease, and in a sufficient quantity to prevent the peculiar fermentation in the blood induced by the diphtheritic poison, is necessary to the full play of the specific virtues of the remedy; but, on the contrary, that of quinia at a later stage and in a moderate dose to sustain the nerve-centres and arouse the latent energies of the system. Nevertheless, to intensify the power of alcohol by imparting through the nerves more vitality to the blood, it is safer in bad cases to inaugurate the treatment with both, and, in this way, anticipate the depression that soon follows the appearance of the membrane.

The office of iron is much inferior to that of quinia. It is not to fight the battle but to complete the victory; not to defend the citadel but to rebuild its shattered walls; and yet, in order to remove every evidence of the terrible struggle, and restore the vital forces to their wonted supremacy, the aid of alcohol is needed more or less constantly.

To show the sufficiency of these three agents, unaided by others, medical or surgical, I will here append the histories of certain typical cases which illustrate both the routine of my treatment, and its success in the most desperate straits.

A boy three years old was taken in the evening with croup. At first emetic, and then nauseating doses of ipecacuanha were given, but in the morning, to head off any lurking tendency there might be to the membranous form, fifteen drops of whiskey every two hours. The third day, the mother had a small diphtheritic patch on one tonsil. Two teaspoonfuls of whiskey and one grain each of quinoidine and sulphate of cinchonia were directed every two hours. The whiskey ordered for the boy was now given to his brother, two years younger, in ten drop doses. On the fourth day the first, and on the eleventh the second son, were attacked with scarlatina in a mild form. The alcoholic treatment was followed without addition until convalescence was established. On the twelfth day the daughter, twenty-one years of age, the only remaining member of the family was attacked with a raging fever, intense headache, and membranous inflammation of both tonsils. In her case, the same medicines were prescribed as those taken by the mother. All recovered promptly, and suffered from no secondary disorder; except the mother who had, some ten days after the fall of the membrane, a dimness of vision that forbade reading, sewing, or other continuous application of the eyes. This symptom necessitated a return to the original treatment for a couple of weeks.

A gentleman who had for many years been superintendent of a large brass manufactory, where his nerves had at length become so shattered by the roar of machinery and the reverberation of metal that he was about retiring to a farm in Connecticut, was taken down with pneumonia and diphtheria simultaneously. Brandy and quinine carried him quickly and safely through these diseases; whereupon the country air, imparting tone to the nervous system, restored the full vigor of former years.

A child seventeen months old, badly nourished, and subjected to the adverse conditions of the back basement of a tenement house, came under my care the third day of its illness. The exudation covered the fauces, and filled the nostrils. The voice was extinct, the head thrown back, and the inspiration laborious and whistling. I held out no hopes to the parents of recovery, but told them that their only chance was to give, as long as the child could swallow, the following prescription.

| | | |
|---|---|---|
| ℞. Quiniae sulp. . . . . . | gr. vi. | |
| Acid. sulp. aromat. . | qtt xx. | |
| Sp. frumenti. . . . . | ℥i. | |
| Aq. fontanae . . . . | ℥ij. | M. |

S. A teaspoonful every hour and a half in water.

This was given singly and uninterruptedly until convalescence was established, when the whiskey alone was relied upon.

On the third day of my attendance, as the membranous secretion gave place to a purulent one, and the respiration improved, the ears became filled with the same deposit, and the cervical glands enlarged by a low form of inflammation. Two of these glands quickly suppurated and were lanced. In the mean time the exudation having disappeared, the medicine was given at longer intervals and, lime-water and condensed milk substituted for the mother's milk, which was watery and insufficient.

In a day or two the child began to struggle for breath, as in membranous croup, when the quinine and whiskey were re-ordered as at first. The effect was a copious purulent secretion, a disintegration of the membrane, and the relaxation of the spasm. As soon, however, as this danger was past, a graver one, if possible, presented itself—the extension of the exudation to the bronchial tubes. The child did not expand its chest, had a livid, bronzed look, was bathed in a clammy, profuse perspiration, and felt as cold as a piece of marble.

I directed an emetic of ipecacuanha, sinapisms to the chest, dry warmth to the extremities, and a continuance of the prescription. The emesis, bringing up a large amount of muco-purulent matter, gave instant relief. Afterwards, the child vomited once or twice a day spontaneously. My attendance extended from February 19th to March 11th; but ten days before the last date, the recovery was assured. This was not retarded, or marred by any secondary trouble whatever.

A boy six years of age, was taken with diphtheritic sore throat; and, though the local disease remained much the same, he rapidly grew worse. His blood seemed saturated with the poison; as shown by the stomach rejecting all food and drink, by the heart acting feebly and irregularly, by the extremities being cold and shrivelled, and the skin having a dusky, sodden look. At first, one and then two teaspoonfuls of brandy were given every two hours; but soon the larger dose every hour. Food in any form would not remain on the stomach; and quinine, promptly rejected, induced a retching that was slow to subside. The brandy, only tolerated in divided doses, had to be often repeated to make good the loss. On the third day, there was a typhus-like fever; lividity of the surface, oppression of the brain, muttering delirium, and a quick, feeble pulse. On the fourth day scarlatina made its appearance. The eruption, save mottled blotches on the face, was confined to the trunk, and had a deep raspberry hue. As the symptoms assumed greater gravity, the brandy was given in three drachm doses every hour, and aided, as far as the stomach would permit, by quinine, and lime-water and milk. At the advent of the eruption, the membrane began to spread over the fauces and extended up the posterior nares, and, at its decline, to be detached in putrid flakes and discharged in purulent sputa. The active treatment extended over two weeks, and resulted without accident in a speedy and perfect recovery.

A girl, nine and a half years old, deficient in constitutional force, weakened by too rapid growth, and afflicted with lateral curvature of the spine, was taken with diphtheria. The exudation on the third day covered the fauces, filled the nostrils, and extended along the roof of the mouth. The symptoms became more and more typhoid, and the putrescence more and more pronounced. Food and drink were not tolerated, quinia induced violent retching, and brandy was vomited unless given in small quantities. The case seemed desparate, as it presented all the signs of malignancy. Beginning at first with smaller doses, the brandy was soon increased to four drachms the hour, and when rejected was made good. As the stomach became more tractable, lime-water and milk, scalded in a thin decoction of farina, was given for nourishment; as the disease began to yield quinia was administered in suppositories; and, as the membrane lost its hold and was supplanted by a purulent secretion, the tincture of iron in small and frequent doses was conjoined with the brandy. In two weeks convalescence was fully inaugurated; and in three, when the local disease was completely subdued, a semi-paralytic state of the heart showed itself, and necessitated the removal of pillows and a rigid enforcement of the horizontal posture. Eventually, she was restored to her usual health without a drawback, excepting a discharge from the right ear.

On the fifth day of this patient's illness, two of her younger sisters were attacked with scarlatina. The whiskey which they, together with the other members of the family, had been taking as a prophylactic was now continued as a medicine. The only addition was a diaphoretic mixture. They passed through the fever in the most satisfactory manner, it being neither aggravated by complications, nor prolonged by secondary disorders. The others in the house, some twelve in number exclusive of the servants, escaped by the virtues of the antidote, though the scraping of walls, the burning of clothes, the tearing up of carpets, etc., were omitted.

I was called on a Sunday evening by Dr. ——, a friend of mine, to see a lady whose child had died the morning previous of diphtheria. She had held the child most of the time of its illness, and kissed it repeatedly and frantically on the lips, as prutrescent matter flowed from them in its last moments. Friday and Saturday she had had an exudation of moderate extent in the fauces, but now in addition a diffused intumescence and induration of the neck. Seeing that the patient had inhaled the child's breath for six days, had had patches on both tonsils for two, and had applied her mouth to the poison many times, I mentioned to the Doctor that we had all the conditions present, foreshadowing a malignant case, and that, regarding alcohol as a true antidote, I would advise its administration in full doses, the same as when the disease was at its height. Besides the quinine previously prescribed, the patient took a tablespoonful of whiskey every hour with this result; she attended the child's funeral on Tuesday, and in a day or two regained her usual health. In this case, the abortive power of alcohol was fully manifested—a power it rarely fails to assert.

To further substantiate the virtues of alcohol in the treatment of diphtheria, I will in conclusion appeal to the records of the Health Board of this city. From the figures furnished me by two persons connected with this department, it appears:

In 1874 there were reported 1651 cases of diphtheria; in 1875, 2669 cases, and in 1876, 2329 cases; making in all 6649 cases.

During the same years, respectively, there were reported 580, 965, and 810 deaths from diphtheria, and 318, 451, and 412 deaths from croup, making the total mortality from diphtheria 2355, from croup 1181, and from diphtheria and croup 3536.

Now if it be assumed that all the cases reported as diphtheria were genuine, and that the majority of deaths from croup had a diphtheritic origin, as doubtless is true, then there will be slight grounds for congratulation over the progress made by therapeutics. The mortality is truly appalling. Even including the croupous, among the diphtheritic cases the exhibit is far from flattering.

From Jan. 10th, 1874, when I lost a young girl ten years of age, to this date Sept. 15th, 1877, I have reported 78 cases of diphtheria and lost one only. This even does not rightfully belong to the list, as the child had been under the care of a homœopathic physician several days. A younger child lay dead in the house when I was called. Beside, the patient refused all food and medicine, and died in thirty-six hours. An older child promptly recovered.

To these 78 cases should be added some eight cases, or more, which, from the slight local trouble or other cause were not reported; but which, from the course of the disease, I am convinced were really diphtheritic. Three of these cases, from the delay in resorting to brandy and quinine at the outset, required much longer treatment than usual. Thus it appears that, in a period of three and a half years, 85 cases of diphtheria have been treated successively without a failure. What is more, not a death appears under the head of croup, or other disease, the sequel of the diphtheritic poison.

The following report was furnished me as to the cases and causes of death—old age, 2; heart disease, 2; softening of brain, 1; cancer, 1; apoplexy, 1; phthisis, 2; preumonia, 1; congestion of lungs, 1; jaundice, 1; typhoid fever, 1; cerebro-spinal meningitis, 1; cystitis, 1; Bright's disease, 1; puerperal fever, 1; diphtheria, 1; scarlatina, 1; cholera infantum, 5; marasmus, 2; convulsions, 2; chronic eczema, 1; whooping cough, 1; and dentition, 2.

The above success, verified beyond cavil, was due almost wholly to the systematic use of whiskey or brandy in definite doses and at fixed intervals, whether the cases were suspicious, mild, or well-marked. A croupy child, if not speedily relieved by the usual remedies, was treated in the same way; as also other members of a family, when one of them had decided diphtheritic symptoms.

Several of my medical friends who have been induced to make trial of the alcoholic treatment, assure me that it gives far better results than any other they had previously employed. Should other members of the profession, from the facts presented in this article, do me the like honor, I am certain they would soon meet diphtheria without fear, treat it with confidence, and attain a success surprising to themselves.

